# Abstract of the Proceedings of the Buffalo Medical Association, August 4, 1874

**Published:** 1874-09

**Authors:** Joseph Fowler

**Affiliations:** Secretary, Pro. Tem.


ART. III.—*Abstract of the Proceedings of the Buffalo Medical Association.—Buffalo, August 4th,* 1874. Reported by JOSEPH FOWLER, M. D., Secretary pro tem.

The Association met at their rooms in the Medical College building, at the usual hour.

The President, Dr. White, in the chair.

Members present: Drs. White, Samo, Gay, Rochester, Bartlett, Brecht, Shaw, Gould, and Fowler. In the absence of the permanent Secretary, on motion, Doctor Fowler was made Secretary pro tempore.

Dr. E. N. Brush was, upon a vote being taken, elected a member of the Association.

Dr. GAY said there was present by request, a patient from the General Hospital, upon whom he had operated for disease of the elbow joint.

The patient had kindly consented to come here to night that he might show the results of resection of the joint, and he requested Dr. Bartow, the resident physician at the Hospital to recite the history of the case, as he was familiar with it.

Dr. BARTOW. Philip Steinmetz, aet. 53, laborer; on election day, Nov. 8th, 1873, patient fell upon a heap of stones striking upon the right elbow, cutting and contusing it. An abscess formed upon the back of the arm just above the elbow, subsequently involving the joint, causing thereby increased inflammation, followed by sloughing of the parts—at the back part and above the elbow for a space of four inches long by two inches in breadth.

The articular surfaces of the bones were exposed and found to be in an ulcerated condition. The whole limb was greatly œdematous and wholly powerless.

The patient had been an inmate of the Erie County Hospital from the time of the receipt of the injury to March 4th, of the present year, when he was removed to the Buffalo General Hospital. Resection of the elbow joint was made by Dr. Gay, March 16th. The longitudinal operation being chosen. The heads of all the bones being removed. The wound was left open—the limb being placed in a tin gutter properly padded. After four weeks, passive motion, supination and pronation were begun; a joint being made in the gutter, corresponding to the elbow joint, by means of which the forearm could be flexed and extended in a very gradual manner. The ulcerated surface and œdematous condition of limb preventing patient from being out of bed.

May 15th, the patient was out of bed and about with his arm in a sling. Was directed to make active motion, and was soon able to partially flex fore arm, carry hand to opposite shoulder, to head, etc. Was soon able to carry a small weight in the hand—this being daily increased until in the course of a month he could carry twenty-five pounds with ease. The œdema slowly disappeared, cicatrix remaining firm. Motion and strength of limb continually improving. The measurement from acromion to styloid process of radius shows an inch of short ening.

At present time, patient is able to do manual labor with nearly as much facility as ever.

Dr. White referred to this operation as one of the greatest achievments of modern, conservative surgery. During his earlier professional life, it was believed that resection of the large joints was necessarily fatal, now it is done with great success.

The patient was examined by the President and members present, the usefulness of the arm and joint tested and the result of the operation pronounced successful.

Dr. Bartlett related the following case as unique and interesting.

A healthy man, 42 years of age, employed as a heater in the Union Iron Works, and possessed of a full head of hair, with whiskers thick and long.

Eight months ago while in the enjoyment of good health, he first noticed his whiskers and the hair on his head beginning to fall off—then from the axillæ pubes, and lastly from the extremities, till none was visible on any part of the body. The eyelids and eyebrows were all gone, not one remaining.

No form of sickness was present when this change was going on. Patient said he never had any severe attack of sickness whatever either general or specific, but on the contrary always enjoyed excellent health.

Dr. ROCHESTER asked whether the color of the hair underwent a change before falling off.

Dr. BARTLETT, replied that it did not, the change taking place in six or eight week from the first noticable disappearance of the hair, and at present there are no indications of its reappearance.

Dr. BARTLETT was asked whether the patient sweat freely when at work? To which he replied in the affirmative.

Dr. ROCHESTER said it might be due to disease of the hair follicles, which it would be well to examine with the lens, and that his occupation might stand in a causative relation to it, as he was subjected to extremes in temperature.

Dr. WHITE said he could not recall a similar case, and would suggest that Dr. Bartlett keep the case under observation, and report at some future meeting, his condition, or any change that might take place, and bring the patient here for examination.

Dr. SAMO related a case of secondary syphilis accompanied with a severe sore mouth, copiously studded with syphilitic eruption which did not yield to his usual plan of treatment. The tongue is fissured and nodulated. Have cauterized the sores and used various applications without being followed by any permanent good. There was a chancre on penis which readily healed after simply cauterizing it. Did not give any medicine internally during this stage, my patient apparently doing well.

There was a red spot on the roof of the mouth, one on the left tonsil and root of the tongue, which did not yield to the local use of nitrate of silver, but on the contrary seemed to be aggravated by it. There were ulcerations on the lips as large as a three cent piece. Sedative applications in the form of washes gave the most relief. The eruption showed itself one week before the mouth began to get sore.

The appetite remained good, throughout the disease, but the patient was unable to take anything but the simplest form of diet.

There was a bubo which did not suppurate. During secondary stage gave mercury iodide combined with five to eight grains of potassium-iodide three times a day. The deuto-iodide of mercury was given in doses of one-sixteenth of a grain ter in die, and continued three weeks.

Dr. WHITE said that in his early practice, he had been called to treat a great many cases of syphilis, and relied on the use of mercury, more frequently in the form of the proto-iodide than any other. He said he was in the habit of prescribing the following combination: hydrar, proto-iod. gr. $\frac{3}{4}$, Conii. gr. jj. in pill three times daily, often continuing their use till gentle constitutional effects have been manifest for a considerable period. Think washes are of little value. Think the eruption may disappear if no medicine whatever be administered.

The deuto-iodide of mercury or corosive sublimate dissolved, in small doses long continued, would be useful in the case under consideration with baths and nourishing diet.

Dr. ROCHESTER: Have never been satisfied that we can successfully treat syphilitic cases without mercury.

Have noticed that most forms go on to tertiary stage. Soft chancre usually heals by one or two applications. Diet and tonics just as important as mercury. Think sedative applications best. By combining deuto-iodide of mercury with potassium iodide a portion of the mercury is eliminated from the system which may prevent its constitutional effects Should add iodide of potassium enough only to dissolve the mercury. Think this may account for the failure in Dr. Samo's case.

Dr. GOULD: I usually rely on the proto-iodide of mercury combined with opium or conium. Obtain good results from the use of bichloride, and proto-iodide of mercury.

Don't think much of the use of caustics; chancre usually heals without applications.

Think local treatment of secondary importance; must rely on constitutional measures.

I have been successful with the use of iodide of mercury in cases of unusual severity.

Dr. BARTLETT said he was in the habit of treating the disease as described. Do not cauterize very much. In advanced cases have more faith in bichloride of mercury than any other form. I have rather a singular case under observation. The patient, a boarding-house keeper, sent for me to treat what she called a felon on her thumb.

The sore looked suspicious, but I could gain no knowledge of the transmission of syphilis unless by contact. On inquiry my patient said she had done washing for a boarder whom she knew to have a disease of a private nature, but did not remember of ever having any abrasion on the thumb. The sore was dusted with calomel, and dressed with the ung. ammon. hydr. and hydr. proto-iodide in half grain doses taken internally three times a day, and under this treatment thumb seemed to get well.

Three or four weeks from that date an abundant eruption of a well marked syphilitic character appeared all over the body.

Dr. Gay agreed with Drs. White and Bartlett. He treated with mercury proto-iodide, giving one-half to one grain three times daily, without opium if the bowels retain it. If they do not, give it night and morning only.

Have no confidence in potassium iodide alone, unless preceeded by mercury iodide in primary or secondary stages. Think the preparation of mercury Dr. Samo used must have been inert or his patient would have improved under its use.

Dr. Samo: It is my custom almost invariably to use mercury in some form from the commencement of the disease, generally using the deuto-iodide or proto-iodide. In combination with potassium iodide I have been very successful in the treatment of secondary symptoms.

Dr. White said he was in the habit of using deuto-iodide of mercury with potassium iodide in the third stage, and proto-iodide with conium in the first and second stages.

Dr. Gay remarked that there was a difference between hard and soft chancre; would recommend for Dr. Samo's case Donovan's solution.

Dr. Rochester: A hard indurated chancre was difficult to cure. Soft chancre was not non-infectious. Have tested it to my sorrow. Am glad to notice that recent writers are beginning to notice the fact that constitutional symptoms will often be developed after them. I have in my mind now the case of a druggist who came to consult me with iritis. Three or four weeks from that date a copious eruption appeared all over his body. He denied having been exposed, but said he had a soft chancre ten years before which got well under the use of simple applications.

Think it many times developes years after the local sore was present. Could relate other incidents of a similar nature.

Dr. SAMO thought it safer to cauterize when first seen, and if done early in many cases nothing further is necessary to effect a cure.

Dr. SHAW used blue mass in preference to other forms of mercury, and iodide of potassium in tertiary stage.

Dr. GAY asked if Dr. Rochester was positive that the patient referred to did not have a chancre more recent than the one already spoken of.

Dr. ROCHESTER said he had not, and that he had seen five or six other cases within three or four years where constitutional symptoms were present.

Dr. GAY said he now had a case under treatment which he regards as syphilitic and which yields to specific treatment, the local lesion being upon the limb.

There are no suspicious circumstances connected with its transmission unless by contact.

Had another similar case where a sore on the face seemed very obstinate to cure but readily healed under the use of syphilitic remedies.

PREVAILING DISEASES.—Scarlet Fever prevails to some considerable extent in the eastern part of the city, but of a less malignant character than before; some cases dying with diphtheria. Whooping cough, cholera-infantum and some diarrhœ also prevailed.

Under this head a discussion arose with reference to the treatment of Scarlatina and its sequelæ, in which Dr. Bartlett said he had adopted a new plan of treating Otitis, and so far had met with good results.

It was to apply a blister on the arm at the point usually selected for vaccination, to be kept in a discharging condition for two or three weeks, if possible, until the ear ceases to discharge, then the blister may be allowed to heal. Some thirty cases submitted to to this treatment are apparently cured.

Dr. WHITE recognized the importance of pure air. This element he believed to be one of the most valuable auxiliaries and the most difficult medicine to obtain.

He used mustard baths when the eruption appeared imperfectly—when the skin was livid and the capillary circulation imperfect.

Hot baths he regarded as excellent also. Think they will bring out the eruption when not well defined. Had seen the entire body annointed with spirits of turpentine, but was unable from his limited observation of its use to speak of its utility.

Dr. Bartlett: Think bathing is often badly employed and a chill follows. To my patients under 5 or 7 years of age I apply the best English mustard in the form of a paste, made by the addition of water only, to an extremity at a time, rubbing it thoroughly and immediately drying with a towel till the skin is of a bright rose color throughout. The patient does not complain; the applications seem to have rather a soothing effect than otherwise. If the rash does not make its appearance in the course of two or three hours the mustard is re-applied, followed by friction.

If eruption does not come well to the surface in four to six hours after, bathe body, portion by portion, with saturated solution of chloride sodium in water.

I anticipate any throat complication by using a flaxseed poultice, covering anterior and lateral portions of the neck. As a result, enlargement of the glands or soreness of the fauces has been exceedingly rare.

Iodine and other applications I believe to be injurious rather than beneficial.

I have tested the salivary secretions, perspiration, tears, urine and alvine evacuations and find them intensely acid, and reason from this that the disease is to be treated by essentially alkaline medicines.

Believe preparations of iron injurious. Have given no medicine whatever in the last one hundred cases treated.

Had one case where temperature reached 107° F. To this patient I applied cloths, wrung out in cold water, to the trunk, noting the decline of temperature, desisting from the application when the temperature was moderately reduced.

Eruption did not recede. Desquamation being established as early as the fifth day. Patient recovered without any of the ordinary sequelæ of scarlet fever.

The doctor promised to perfect his observation and make it the subject of a special paper to be read before the Association at some future meeting.

On motion of Dr. Rochester meeting adjourned.

